# Scattering of Magnetoacoustic Waves and Dynamic Stress Concentration around Double Openings in Piezomagnetic Composites

**DOI:** 10.3390/ma14226878

**Published:** 2021-11-15

**Authors:** Huanhuan Xue, Chuanping Zhou, Gaofei Cheng, Junqi Bao, Maofa Wang, Yongping Gong, Huawei Ji, Wu Yang, Bo Hou, Weihua Zhou, Qiaoyi Wang, Jing Ni

**Affiliations:** 1 School of Mechanical Engineering, Hangzhou Dianzi University, Hangzhou 310018, China; weiyangchaohu@163.com (H.X.); gyp@hdu.edu.cn (Y.G.); jhw76@hdu.edu.cn (H.J.); 202040123@hdu.edu.cn (W.Y.); wangqiaoyi1989@hdu.edu.cn (Q.W.); nj2000@hdu.edu.cn (J.N.); 2 Hangzhou Changchuan Technology Co. Ltd., Hangzhou 310018, China; chenggf@hzcctech.net (G.C.); baojq@hzcctech.net (J.B.); 3 Key Laboratory for Technology in Rural Water Management of Zhejiang Province, Hangzhou 310018, China; houbo1985@163.com; 4 College of Electrical Engineering, Zhejiang University, Hangzhou 310027, China; dididi@zju.edu.cn

**Keywords:** piezomagnetic composites, double openings, magnetoacoustic wave, local coordinate method, dynamic stress concentration

## Abstract

Based on the magnetoacoustic coupled dynamics theory, the wave function expansion method is used to solve the problem of acoustic wave scattering and dynamic stress concentration around the two openings in e-type piezomagnetic composites. To deal with the multiple scattering between openings, the local coordinate method is introduced. The general analytical solution to the problem and the expression of the dynamic stress concentration are derived. As an example, the numerical results of the dynamic stress distribution around two openings with equal diameters are given. The effects of the parameters, such as the incident wave number and the spacing between the openings, on the dynamic stress concentration factor are analyzed.

## 1. Introduction

Piezomagnetic materials can achieve rapid response due to their direct magnetoacoustic conversion characteristics. Piezomagnetic materials can be widely used in the production of sensors, resonators, filters, retarders, controllers, and other components and intelligent systems. Therefore piezomagnetic materials play an important role in the industry, information, energy, aerospace, biomedical, defense weaponry, and civil engineering fields [1,2,3,4,5]. Additionally, piezomagnetic materials in the production process will inevitably occur within defects. Sometimes holes need to be opened in order to meet the needs of the process in the materials. These defects or artificial holes can cause geometric discontinuities. Defects or holes near the stress concentration phenomenon will greatly reduce the piezomagnetic material structure of the load capacity and reduce the structure of the service life. Therefore, it is very important to study the dynamic stress concentration of piezomagnetic materials with defects or holes.

There are many scholars committed to the study of piezomagnetic materials in the application of elastic materials. There are relatively few studies on the fracture behavior of piezomagnetic materials and the dynamic behavior of stress concentration caused by holes. The traditional boundary conditions of the cracked surface of a piezomagnetic material containing microcracks have been well defined, and a more mature conclusion has been obtained: the far field problem of the scattering of SH waves from the cracks between the cylindrical inclusions and the piezomagnetic bodies is studied. Jiao et al. [4] studied and analyzed a large number of calculations. Materials containing noncrack initial defects can produce stress concentration under external loads. Liang et al. [5] studied the stress intensity factor at a crack tip in a two-dimensional anisotropic piezomagnetic plate with a hole-edge crack by using the method of mixed boundary element, and gave a numerical solution of the problem. Then, the same method was used to study a hole crack in an elastic-electric-magnetic coupling composite plate, and a numerical solution of the stress intensity factor at the crack tip was given [6]. Wang and Mai [7] used the integral transformation theory to study the dynamic response of cracks in a piezomagnetic material under the impact of the magnetic field and the antiplane load in any plane, and the stress concentration intensity of the problem and the dependence of the magnetic field and displacement field were obtained. Cao et al. [8] carried out a theoretical research on the dynamic antiplane problem of two crack interactions in a piezomagnetic material. Singh et al. [9] used the integral transformation technique and the Cauchy singular integral equation to study the scattering of the elastic wave at the interface between the elastic matrix and the piezomagnetic layer and discussed the effects of different incident wave angles and material combinations on it. Tian et al. [10] conducted an in-depth study of the multiple scattering of the magnetoelastic wave and dynamic stress around a buried cavity in a functional gradient piezomagnetic material layer combined with a uniform piezomagnetic material. Wang et al. [11] studied the dynamic stress concentration in a piezoelectric material with a noncircular hole subjected to an SH wave by using the mapping method. Zhao et al. [12] studied the propagation of elastic waves in a bilayer composed of a piezomagnetic layer and a piezomagnetic layer. Pang et al. [13] studied the influence of the incident wave on the dynamic stress concentration factor around a cylinder in a 1–3 type piezomagnetic composite material, and studied its piezomagnetic properties. Zhang et al. [14] studied the two arbitrary shapes of voids in infinite piezomagnetic solid under uniform mechanical and magnetic loads and gave two-dimensional perturbation solutions. Kong et al. [15] used Green function method to study the SH wave scattering and dynamic stress intensity factor in an infinite piezomagnetic material with a radial finite length crack. They got the edge of the hole dynamic analytical expressions for stress intensity factors. Sahu et al. [16] established an analytical method for solving the scattering of SH wave and dynamic stress concentration in the vicinity of the interface of a semi-infinite piezomagnetic material. In light of the solution to boundary value problems describing wave scattering by cracks in an infinite plane region, a non-hypersingular traction boundary integral equation method is developed.

In the integral transformation method, seeking points in the inverse transformation process is sometimes very difficult. Additionally, this method requires the function to meet certain conditions, so it has a large limitation. With the development of the computer, numerical methods are used to solve the stress calculation problem of a piezomagnetic material discontinuous structure under a dynamic load. While these numerical methods are very useful, it is equally important to understand the physical properties through theoretical analysis.

In this paper, we will further seek to solve the problem of scattering and dynamic stress concentration in the elastic wave around the double openings in a piezomagnetic medium. The numerical results of the dynamic stress distribution around the openings are given, and the influence of the parameters, such as incident wave number and interopening spacing, on the dynamic stress concentration factor is analyzed.

## 2. The Incidence of Magnetoacoustic Wave and the Total Magnetic Wave Field

In this paper, the governing equations and constitutive relations of the steady-state antiplane dynamics problem in the piezomagnetic body are controlled in the piezomagnetic bodies. The governing equations of the steady-state antiplane dynamics problem are:

(1) $$\begin{array}{l} \frac{\partial B_{x}}{\partial x}+\frac{\partial B_{y}}{\partial y}=0\\ \frac{\partial \tau_{xz}}{\partial x}+\frac{\partial \tau_{yz}}{\partial y}=\rho \frac{\partial^{2}w}{\partial t^{2}} \end{array}$$

where $\tau_{xz}$ and $\tau_{yz}$ are shear stress components, $B_{x}$ and $B_{y}$ are the magnetic flux densities, and ρ is the mass density. The constitutive relationship of piezomagnetic materials can be written as:

(2) $$\begin{array}{l} \tau_{xz}=c_{44}\frac{\partial w}{\partial x}+h_{15}\frac{\partial \phi }{\partial x}\\ \tau_{yz}=c_{44}\frac{\partial w}{\partial y}+h_{15}\frac{\partial \phi }{\partial y}\\ B_{x}=h_{15}\frac{\partial w}{\partial x}-\kappa_{11}\frac{\partial \phi }{\partial x}\\ B_{y}=h_{15}\frac{\partial w}{\partial y}-\kappa_{11}\frac{\partial \phi }{\partial y} \end{array}\}$$

where $c_{44}$ is the elastic constant of the piezomagnetic material, $h_{15}$ is the piezomagnetic constant of the piezomagnetic material, $\kappa_{11}$ is the magnetic medium constant of the piezomagnetic material, and ϕ is the potential in the medium.

**Figure 1:**
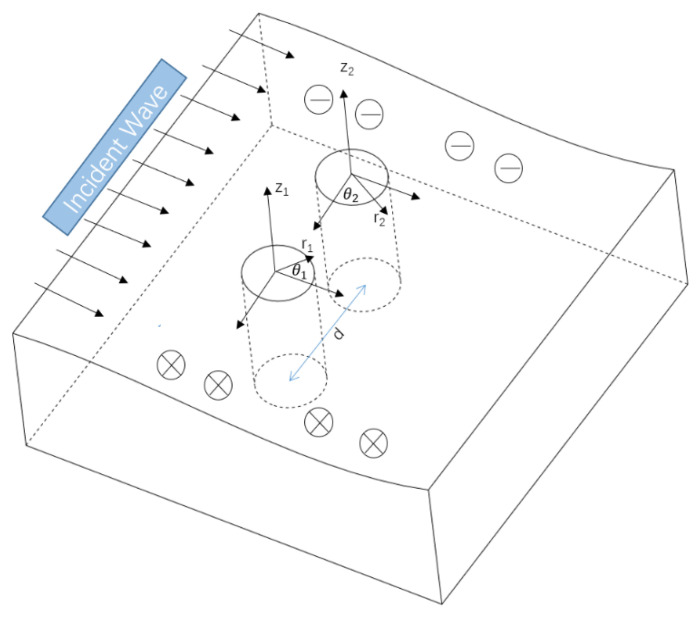
Elastic wave incident in piezomagnetic composites with double openings.

Considering an infinite piezomagnetic material with two openings, a steady-state SH wave is incident along the *x*-axis direction shown in Figure 1. Additionally, the time factor is omitted, and the corresponding out-of-plane displacement field $w^{\left(i\right)}$ and the potential field $\phi^{\left(i\right)}$ in the plane can be expressed as:

(3) $$\begin{array}{l} w^{\left(i\right)}=w_{0}\sum_{n=-\infty }^{\infty }i^{n}J_{n}\left(k\left|\Omega \left(\eta \right)\right|\right){\left\{\frac{\Omega (\eta )}{\left|\Omega (\eta )\right|}\right\}}^{n}\\ \phi^{\left(i\right)}=\frac{h_{15}}{\kappa_{11}}w^{\left(i\right)} \end{array}\}$$

In the analysis and calculation, the local coordinate system can be used to convert the internal force component in each local polar coordinate system to the polar coordinate system to be calculated. Considering the multiple scattering between the openings, the acoustic wave scattering field generated in the m opening in the polar coordinate system $\left(r_{m},\theta_{m}\right)$ can be described as:

(4) $$\begin{array}{l} w^{\left(s\right)}=\sum_{m=1}^{2}\sum_{n=-\infty }^{\infty }A_{n}^{m}H_{n}^{\left(1\right)}\left(kr_{m}\right)e^{in\theta_{m}}\\ \phi^{\left(s\right)}=\frac{h_{15}}{\kappa_{11}}w^{\left(s\right)}+\sum_{m=1}^{2}\sum_{n=0}^{\infty }B_{n}^{m}{\left(kr_{m}\right)}^{-n}e^{in\theta_{m}} \end{array}\}$$

where $A_{n}^{m}$ and $B_{n}^{m}$ (m=1,2) are the scattered wave pattern coefficients produced by the first opening, which are determined by the boundary conditions. When the boundary value of the piezomagnetic medium is solved, the total wave field of the antiplane shear wave should be superimposed by the incident field and the scattering field. The total field in the double-opening piezomagnetic material is:

(5) $$\begin{array}{l} w^{\left(t\right)}=w^{\left(i\right)}+w^{\left(s\right)}\\ \phi^{\left(t\right)}=\phi^{\left(i\right)}+\phi^{\left(s\right)} \end{array}\}$$

Then

(6) $$w^{\left(t\right)}=\sum_{n=-\infty }^{\infty }[w_{0}i^{n}J_{n}\left(kr\right)+\sum_{m=1}^{2}A_{n}^{m}H_{n}^{\left(1\right)}\left(kr_{m}\right)]e^{in\theta_{m}}$$

(7) $$\phi^{\left(t\right)}=\frac{h_{15}}{\kappa_{11}}\sum_{n=-\infty }^{\infty }\sum_{m=1}^{2}[w_{0}i^{n}J_{n}\left(kr\right)+\sum_{m=1}^{2}A_{n}^{m}H_{n}^{\left(1\right)}\left(kr_{m}\right)]e^{in\theta_{m}}+\sum_{n=0}^{\infty }\sum_{m=1}^{2}B_{n}{\left(kr_{m}\right)}^{-n}e^{in\theta_{m}}$$

There is no elastic displacement field in the circular opening. Only the potential field $\phi^{c}$ exists, and the charge density is zero. Therefore, the solution should satisfy the Laplace equation $\nabla^{2}\phi =0$. Considering that the potential in the circular opening cannot be infinite, it should be a finite value, so its expression can be written as:

(8) $$\phi^{c}=\sum_{n=0}^{\infty }\sum_{m=1}^{2}C_{n}{\left(kr_{m}\right)}^{n}e^{in\theta_{m}}$$

The corresponding stress can be expressed as:

(9) $$\begin{array}{cc} \tau_{\rho z} & =\frac{kc_{44}}{2}\left(1+\lambda \right)\sum_{n=-\infty }^{\infty }\sum_{m=1}^{2}\{w_{0}i^{n}[\frac{\eta_{m}}{\rho_{m}}\frac{\Omega^{\prime }\left(\eta_{m}\right)}{\left|\Omega^{\prime }\left(\eta_{m}\right)\right|}J_{n-1}\left(k\left|\Omega \left(\eta_{m}\right)\right|\right){\left\{\frac{\Omega (\eta_{m})}{\left|\Omega (\eta_{m})\right|}\right\}}^{n-1}\\ & -\frac{\bar{\eta_{m}}}{\rho_{m}}\frac{\bar{\Omega^{\prime }\left(\eta_{m}\right)}}{\left|\Omega^{\prime }\left(\eta_{m}\right)\right|}J_{n+1}\left(k\left|\Omega \left(\eta_{m}\right)\right|\right)]{\left\{\frac{\Omega (\eta_{m})}{\left|\Omega (\eta_{m})\right|}\right\}}^{n+1}+A_{n}[\frac{\eta_{m}}{\rho_{m}}\frac{\Omega^{\prime }\left(\eta_{m}\right)}{\left|\Omega^{\prime }\left(\eta_{m}\right)\right|}\\ & \times H_{n-1}^{\left(1\right)}\left(k\left|\Omega \left(\eta_{m}\right)\right|\right){\left\{\frac{\Omega (\eta_{m})}{\left|\Omega (\eta_{m})\right|}\right\}}^{n-1}-\frac{\bar{\eta_{m}}}{\rho_{m}}\frac{\bar{\Omega^{\prime }\left(\eta_{m}\right)}}{\left|\Omega^{\prime }\left(\eta_{m}\right)\right|}H_{n+1}^{\left(1\right)}\left(k\left|\Omega \left(\eta_{m}\right)\right|\right)\\ & \times {\left\{\frac{\Omega (\eta_{m})}{\left|\Omega (\eta_{m})\right|}\right\}}^{n+1}]\}-h_{15}\sum_{n=0}^{\infty }\sum_{m=1}^{2}B_{n}nk^{-n}\frac{\bar{\eta_{m}}}{\rho_{m}}\frac{\bar{\Omega^{\prime }\left(\eta_{m}\right)}}{\left|\Omega^{\prime }\left(\eta_{m}\right)\right|}{\left(\bar{\Omega (\eta_{m})}\right)}^{-n-1} \end{array}$$

(10) $$\begin{array}{c} B_{\rho }=h_{15}\frac{\partial w}{\partial r}-\kappa_{11}\frac{\partial \phi }{\partial r}=\kappa_{11}\sum_{n=0}^{\infty }\sum_{m=1}^{2}B_{n}nk^{-n}\frac{\bar{\eta_{m}}}{\rho_{m}}\frac{\bar{\Omega^{\prime }\left(\eta_{m}\right)}}{\left|\Omega^{\prime }\left(\eta_{m}\right)\right|}{\left(\bar{\Omega (\eta_{m})}\right)}^{-n-1}\\ B_{\rho }^{c}=-\kappa_{0}\phi^{c}=-\kappa_{0}\sum_{n=0}^{\infty }\sum_{m=1}^{2}C_{n}nk^{n}\frac{\eta_{m}}{\rho_{m}}\frac{\Omega^{\prime }\left(\eta_{m}\right)}{\left|\Omega^{\prime }\left(\eta_{m}\right)\right|}{\left(\Omega (\eta_{m})\right)}^{n-1} \end{array}$$

where $\lambda =\frac{h_{15}^{2}}{c_{44}\kappa_{11}}$ is a dimensionless piezomagnetic constant, and $\kappa_{0}$ is the magnetic medium constant in vacuum.

## 3. Boundary Conditions and Mode Coefficients for Double Openings

In the η plane, let the openings be free boundary conditions, and six boundary conditions can be given as:

(11) $$\begin{array}{l} \tau_{\rho_{m}z}|{}_{\rho_{1}=a_{1}}=0\\ B_{\rho_{m}}|{}_{\rho_{1}=a_{1}}=B_{\rho_{m}}^{c}|{}_{\rho_{1}=a_{1}}\\ \phi |{}_{\rho_{m}=a_{1}}=\phi^{c}\\ \tau_{\rho_{m}z}|{}_{\rho_{2}=a_{2}}=0\\ B_{\rho_{m}}|{}_{\rho_{2}=a_{2}}=B_{\rho_{m}}^{c}|{}_{\rho_{2}=a_{2}}\\ \phi |{}_{\rho_{m}=a_{2}}=\phi^{c} \end{array}\}$$

where $a_{1}$ and $a_{2}$ are the equivalent radii of two openings.

Equations (6) and (7) are substituted into Equation (11) which are boundary condition. According to the orthogonality of the function system, six mode coefficients, $A_{n}^{1},B_{n}^{1},C_{n}^{1},A_{n}^{2},B_{n}^{2},C_{n}^{2}$, can be obtained by the following equations:

(12) $$\sum_{j=1}^{6}\sum_{n=-\infty }^{\infty }H_{n}^{i}X_{n}=H_{i}\;(i=1,2,3,4,5,6)$$

$$H_{n}=\left[\begin{array}{cccccc} H_{11}^{n} & H_{12}^{n} & H_{13}^{n} & H_{14}^{n} & H_{15}^{n} & H_{16}^{n}\\ H_{21}^{n} & H_{22}^{n} & H_{23}^{n} & H_{24}^{n} & H_{25}^{n} & H_{26}^{n}\\ H_{31}^{n} & H_{32}^{n} & H_{33}^{n} & H_{34}^{n} & H_{35}^{n} & H_{36}^{n}\\ H_{41}^{n} & H_{42}^{n} & H_{43}^{n} & H_{44}^{n} & H_{45}^{n} & H_{46}^{n}\\ H_{51}^{n} & H_{52}^{n} & H_{53}^{n} & H_{54}^{n} & H_{55}^{n} & H_{56}^{n}\\ H_{61}^{n} & H_{62}^{n} & H_{63}^{n} & H_{64}^{n} & H_{65}^{n} & H_{66}^{n} \end{array}\right]$$

$$\begin{array}{l} X_{n}^{}={\left[A_{n}^{1}\;B_{n}^{1}\;\;C_{n}^{1}\;A_{n}^{2}\;B_{n}^{2}\;\;C_{n}^{2}\right]}^{T}\\ H_{i}={\left[H_{1}\;H_{2}\;\;H_{3}\;\;H_{4}\;\;H_{5}\;H_{6}\right]}^{T} \end{array}$$

$\exp(-\mathrm{is}\;\theta_{m})$ is multiplied by Equation (12) and is integrated over the interval (−π,π), and the infinite algebraic equations are given as follows:

(13) $$\sum_{n=-\infty }^{\infty }H_{ns}X_{n}=H_{s}$$

where $H_{ns}=\frac{1}{2\pi }\int_{-\pi }^{\pi }H_{n}\exp\left(-is\theta_{j}\right)d\theta_{j},H_{s}=\frac{1}{2\pi }\int_{-\pi }^{\pi }H_{i}\exp\left(-is\theta_{j}\right)d\theta_{j}.$

## 4. Dynamic Stress Concentration Factor

The dynamic stress concentration is defined by the ratio of the circumferential dynamic stress on the circumference of the opening to the magnitude of the circumferential stress in the incident direction:

(14) $$\begin{array}{cc} \tau_{\theta z} & =\frac{ik}{2}\left(1+\lambda \right)\sum_{n=0}^{\infty }\{[\varepsilon_{n}\frac{\eta_{m}}{\rho_{m}}\frac{\Omega^{\prime }\left(\eta_{m}\right)}{\left|\Omega^{\prime }\left(\eta_{m}\right)\right|}J_{n-1}\left(k\left|\Omega \left(\eta_{m}\right)\right|\right){\left\{\frac{\Omega (\eta_{m})}{\left|\Omega (\eta_{m})\right|}\right\}}^{n-1}\\ & +\frac{\bar{\eta_{m}}}{\rho_{m}}\frac{\bar{\Omega^{\prime }\left(\eta_{m}\right)}}{\left|\Omega^{\prime }\left(\eta_{m}\right)\right|}J_{n+1}\left(k\left|\Omega \left(\eta_{m}\right)\right|\right){\left\{\frac{\Omega (\eta_{m})}{\left|\Omega (\eta_{m})\right|}\right\}}^{n+1}]+\sum_{m=1}^{2}A_{n}^{m}[\frac{\eta_{m}}{\rho_{m}}\frac{\Omega^{\prime }\left(\eta_{m}\right)}{\left|\Omega^{\prime }\left(\eta_{m}\right)\right|}\\ & \times H_{n-1}^{\left(1\right)}\left(k\left|\Omega \left(\eta_{m}\right)\right|\right)\times {\left\{\frac{\Omega (\eta_{m})}{\left|\Omega (\eta_{m})\right|}\right\}}^{n-1}+\frac{\bar{\eta_{m}}}{\rho_{m}}\frac{\bar{\Omega^{\prime }\left(\eta_{m}\right)}}{\left|\Omega^{\prime }\left(\eta_{m}\right)\right|}H_{n+1}^{\left(1\right)}\left(k\left|\Omega \left(\eta_{m}\right)\right|\right)\\ & \times {\left\{\frac{\Omega (\eta_{m})}{\left|\Omega (\eta_{m})\right|}\right\}}^{n+1}]\}+{\mathrm{ih}}_{15}\times \sum_{n=0}^{\infty }\sum_{m=1}^{2}B_{n}^{m}nk^{-n}\frac{\bar{\eta_{m}}}{\rho_{m}}\frac{\bar{\Omega^{\prime }\left(\eta_{m}\right)}}{\left|\Omega^{\prime }\left(\eta_{m}\right)\right|}{\left(\bar{\Omega (\eta_{m})}\right)}^{-n-1} \end{array}$$

## 5. Numerical Examples and Discussion

A steady-state acoustic wave, $w^{\left(i\right)}$, is incident along the *x*-axis, and the circular opening mapping function with the radius $a_{1}=a_{2}=a$ can be taken as:

(15) Ω=aη

Taking the circular opening as an example, the corresponding calculation program is prepared. Here n = 15, the dimensionless wave number is ka=0.1~2.0. CoFe_2_O_4_ is chosen as the materials in the piezomagnetic phase in the numerical examples. The relative material constants are $\rho =5.3\times 10^{3}\;\mathrm{kg}\cdot m^{-3},$ $c_{44}=45.3\times 10^{9}\;N\cdot m^{-2},$ $h_{15}=550\;N\cdot A^{-1}\cdot m^{-1},$ and $\mu_{11}=157\times 10^{-6}\;N\cdot A^{-2}$.

**Figure 2:**
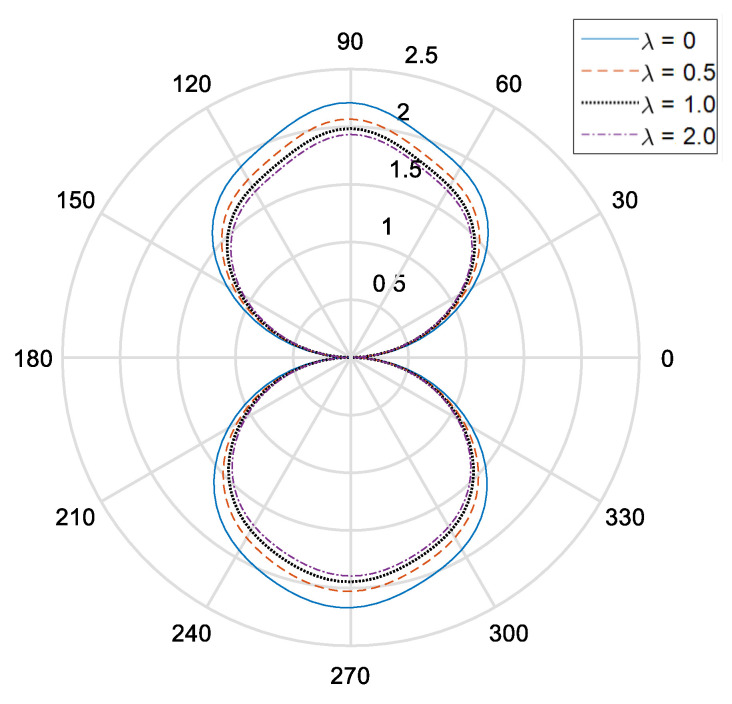
Dynamic stress concentration factor around a single opening in piezomagnetic composites (ka = 0.1, λ = (0, 0.5, 1.0, 2.0)).

**Figure 3:**
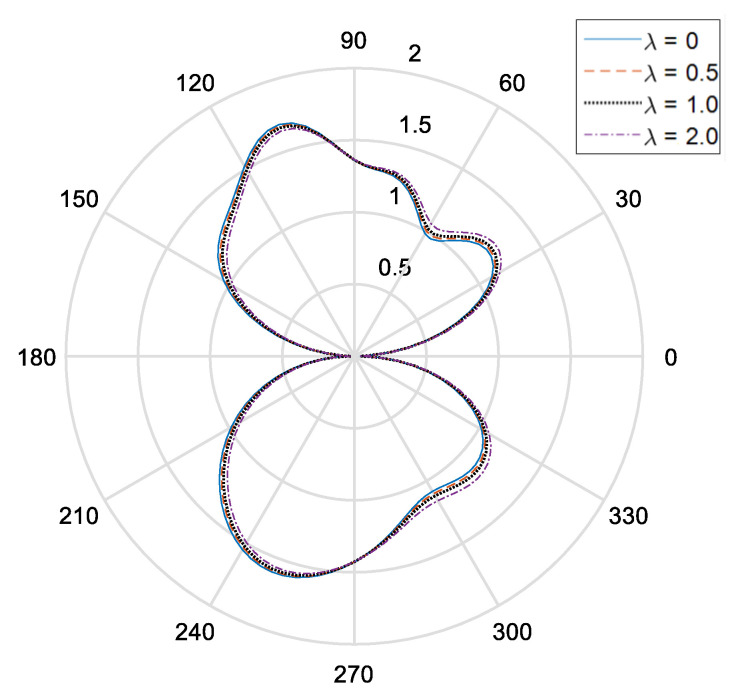
Dynamic stress concentration factor of single opening under different piezomagnetic constants (ka = 2.0, λ = (0, 0.5, 1.0, 2.0]).

**Figure 4:**
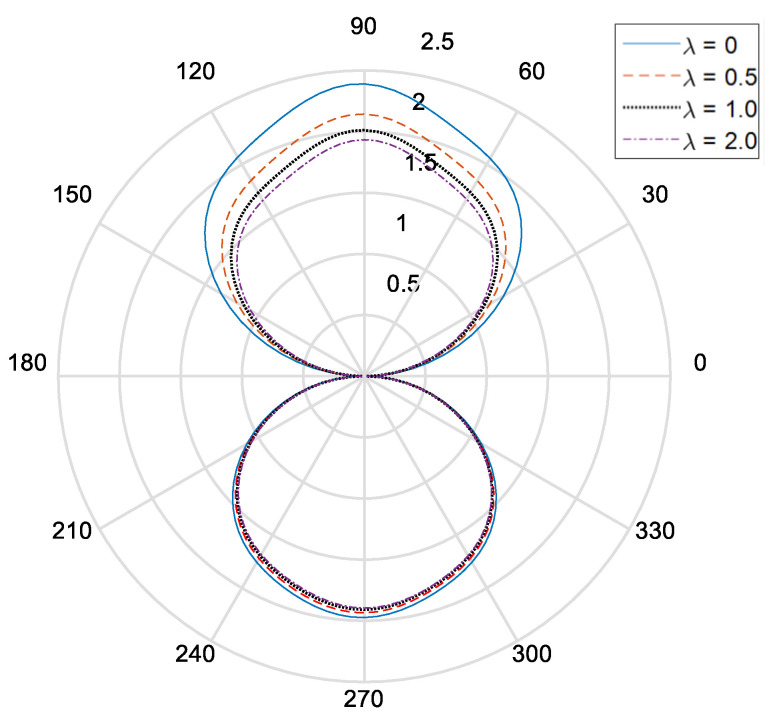
Dynamic stress concentration factor around the upper opening in piezomagnetic composites (ka = 0.1. d/a = 2.1, λ = (0, 0.5, 1.0, 2.0)).

**Figure 5:**
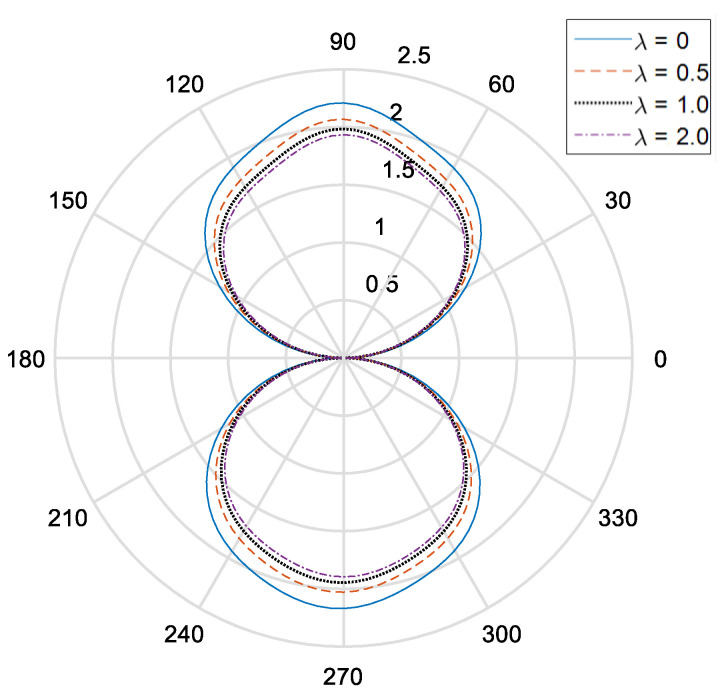
Dynamic stress concentration factor around the upper opening in piezomagnetic composites (ka = 0.1, d/a = 12.0, λ = (0, 0.5, 1.0, 2.0)).

**Figure 6:**
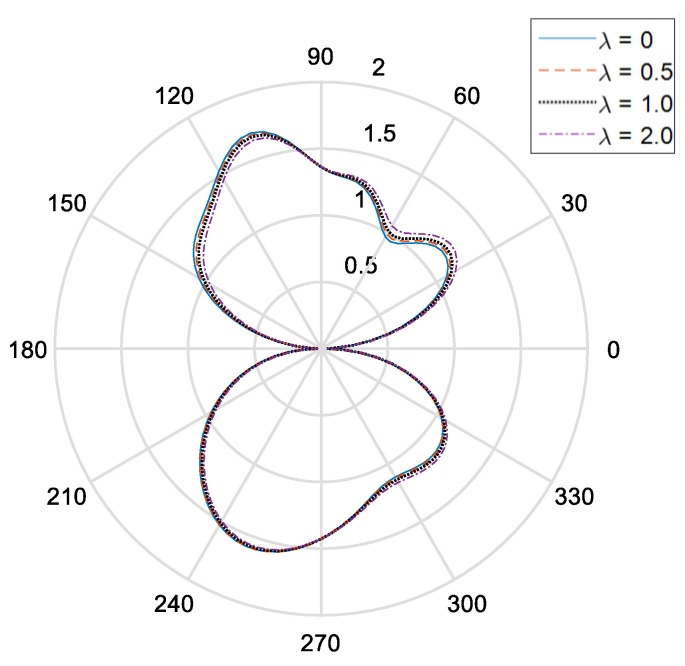
Dynamic stress concentration factor around the upper opening in piezomagnetic composites (ka = 2.0, d/a = 2.1, λ = (0, 0.5, 1.0, 2.0)).

**Figure 7:**
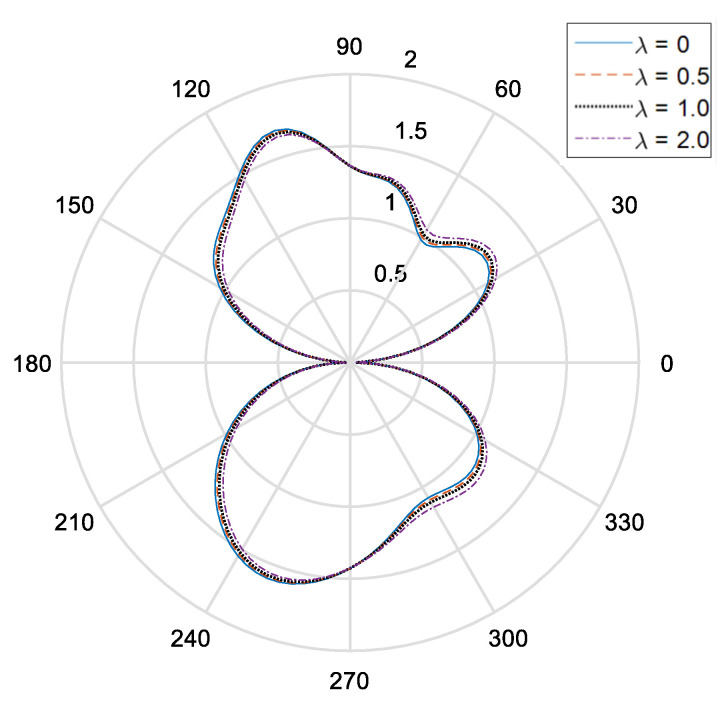
Dynamic stress concentration factor around the upper opening in piezomagnetic composites (ka= 2.0, d/a = 12.0, λ = (0, 0.5, 1.0, 2.0)).

**Figure 8:**
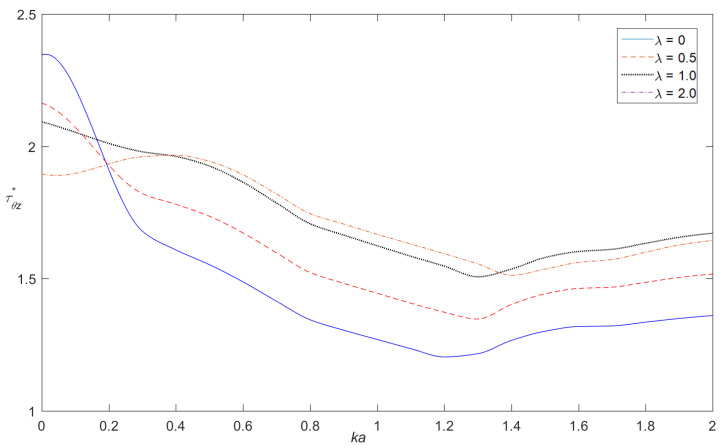
Dynamic stress concentration factor versus incident wave number in piezomagnetic composites with double openings (d/a = 2.1, λ = (0, 0.5, 1.0, 2.0)).

Figure 2 and Figure 3, respectively, show the distribution of the dynamic stress concentrations when the incident wave numbers are ka=0.1 and ka=2.0 under different physical parameters, λ, in the piezomagnetic composites with a single circular opening. Figure 4 and Figure 5 and Figure 6 and Figure 7, respectively, describe the distribution of the dynamic stress concentrations under different physical parameters, λ, with two circular openings when the incident wave numbers are ka=0.1 and ka=2.0. In Figure 4 and Figure 6, the opening spacings are d/a=2.1 (which means that the nearest distance between the two openings is 0.1a). In Figure 5 and Figure 7, the opening spacings are d/a=12.0 (which means that the nearest distance between the two openings is 10.0a). Figure 8 reveals the relationship between the dynamic stress concentration factor and the incident wave number in piezomagnetic composites with the opening spacing d/a=2.1.

It can be seen that when the incident wave frequency is low, the dynamic stress concentrations of the back-wave side (the right half of the figure) and the traveling wave side (the left half side of the figure) are nearly symmetric with respect to the vertical axis. The physical parameter λ has great influence on the dynamic stress concentrations. If the physical parameter λ is great, it will cause a reduction of the dynamic stress concentrations.

The opening spacing also has great influence on the dynamic stress concentrations. When the opening spacing is d/a=2.1, the dynamic stress concentration factor in the top half of Figure 2 is obviously larger than that in the bottom half of Figure 2. It is caused by the wave interaction between the two openings. When the opening spacing is d/a=12.0, the dynamic stress concentration factor in the top half of Figure 5 and Figure 7 is nearly equal to that in the bottom half of Figure 5 and Figure 6, respectively. Nevertheless, there is no such obvious difference in Figure 6 for the reason that the larger incident wave number weakens the interaction between the two openings.

Figure 8 illustrates that a smaller incident wave number causes larger dynamic stress concentrations. When the wave numbers are in the interval of ka=1.2~1.5, there are minimum dynamic stress concentrations.

## 6. Conclusions

In this paper, based on the magnetoacoustic coupled dynamics theory, the scattering of magnetoacoustic coupled waves and dynamic stress concentrations around two openings in the e-type piezomagnetic composites by using the complex function and the conformal mapping method are studied, and the numerical results are given. From the analysis and calculation, results can be seen:

(1) The opening spacing and the physical parameter λ of the e-type piezomagnetic composites have great influence on the dynamic stress concentrations. A great physical parameter, λ, will cause a reduction of the dynamic stress concentrations. It indicates that piezomagnetic parameters increase the hardness of the materials. The opening spacing influences the density of the stress diagram under different physical parameters, λ. When the incident wave number is large, different physical parameters cause the curve spacing greater.
(2) When the wave number is constant, the maximum value of the dynamic stress concentration factor changes with the opening spacing. When the opening spacing is small, the effect of the opening spacing on the dynamic stress concentration factor is very large. When the spacing between the two openings is large, the influence of the spacing on the dynamic stress concentration factor is weakened. When the distance between the two openings is infinite, the dynamic stress concentration factor of the two openings is almost the same as that of the single opening, and the dynamic stress concentration factor is no longer affected by the adjacent opening.
(3) When the opening spacing is constant, with the increasing number of waves, the distance between the two openings on the dynamic stress concentration factor is increasing. The wave incident wave numbers greatly influence the value and distribution of the dynamic stress concentration factors around the openings. In contrast to the solution in the static case, analyses show that the piezomagnetic properties have great effect on the dynamic stress in the region of intermediate frequency. A larger incident wave frequency can enhance the piezomagnetic effects on the dynamic stress concentration.

The theoretical and numerical results of this paper are expected to be applied in the dynamic analysis and strength design of piezomagnetic composites. They can be used to solve the vibration of piezomagnetic composites and structures and determine the vibration high-order mode.

## Data Availability

The study did not report any data.
